# Skin Diseases in a Pediatric Hospital of Nepal

**DOI:** 10.1155/2021/6619936

**Published:** 2021-06-12

**Authors:** Roushan Jahan, Shreedhar Khanal, Shraddha Shrestha, Niraj Parajuli

**Affiliations:** ^1^Department of Dermatology, Kanti Children's Hospital, Kathmandu, Nepal; ^2^Kanti Children's Hospital, Kathmandu, Nepal; ^3^Department of Dermatology, Bharatpur Hospital, Chitwan, Bharatpur, Nepal; ^4^Department of Dermatology, National Academy of Medical Sciences, Bir Hospital, Kathmandu, Nepal

## Abstract

The skin diseases of pediatric population are varied which change according to age and season. There is a rarity of studies on pediatric skin conditions from Nepal. This observational study from the only tertiary care referral pediatric center of the country highlighted the burden of pediatric skin diseases in Nepalese population. All new cases of pediatric patients less than 14 years of age consulting the pediatric dermatological OPD of Kanti Children's Hospital from January 2017 to December 2017 were included in this study. Demographic details of all the patients such as age and sex were recorded. The diagnosis was made clinically in most instances and appropriate laboratory and histopathological examination were performed wherever necessary. A total of 7683 pediatric patients were included in the study. Among these, there were 4574 (59.53%) males and 3109 (40.47%) females. The most common skin condition was infections among 2463 (32.12%) followed by eczematous conditions in 1711(22.27%) and hypersensitivity reactions in 1510 (19.65%). Infections were more common during the summer months. Overall, both infectious and noninfectious skin diseases were significantly more common during the warmer (summer and spring) months as compared to colder (autumn and winter) months (*p* < 0.001). This study shows that the pediatric dermatoses are common in Nepalese population.

## 1. Introduction

Pediatric dermatology is a subspecialty of dermatology that deals with skin diseases in the pediatric population [1] which reflects the status of health and personal hygiene of a community [2]. The worldwide prevalence of pediatric dermatoses is up to 30% [1] and around 22% in Nepal [3, 4].

Similarly, up to 30% of pediatric patients who visit primary care doctors have skin-related problems [5].

The spectrum of pediatric skin diseases varies according to the seasons and geographical locations [3]. Similarly, it also differs according to many environmental and socioeconomic factors. The burden of skin diseases explored through different studies can play a significant role in creating changes in public health and policy making [3]. There are studies showing low quality of life as well as high level mental stress in family members of patients with skin disease [6, 7].

There are only a handful of studies done in pediatric dermatoses in the country. This study aims to elaborate on the patterns of skin diseases in children with respect to the different pediatric age group, sex, and seasonal variations.

## 2. Materials and Methods

This retrospective study was conducted at Kanti Children's Hospital, the only government-run tertiary care central-level pediatric hospital of Nepal located at Kathmandu. It caters to pediatric population of less than 14 years of age. All the details of new patients coming to the pediatric dermatology out-patient department (OPD) of the hospital from January 2017 to December 2017 were analysed. Demographic characteristics of patients including age and sex were obtained from the hospital data. All the diagnoses were made clinically by a single pediatric dermatologist. Laboratory tests were done whenever necessary. Any patients without a clinical diagnosis or missing details were not included. The diagnoses were classified into 18 broad headings. Any of the diseases which did not fit into the mentioned headings were classified as others. The diseases were also classified according to the four seasons, namely, spring, summer, winter, and autumn, in Nepal. They were further grouped according to different age groups, namely, neonates (0–28 days), infants (28 days to 1 year), 1–5 years, 5–11 years, and 11–14 years.

A total of 7683 new patients who visited the pediatric dermatology OPD of the hospital were enrolled in the study. Frequencies of diseases were recorded along with other important demographic details including age and sex.

Frequency and percentages were calculated for all categorical variables and presented in tables.

## 3. Results

A total of 7683 patients visited the dermatology out-patient department at Kanti Children's Hospital during the one year period from January 2017 to December 2017. Males (4574) out-numbered females (3109) with a ratio of 1.47 : 1.

Infections, eczema, and hypersensitivity reactions were the top three pediatric dermatoses which consisted almost two-third of all cases (Table 1). The most common dermatological conditions included infectious dermatosis, eczematous condition, and hypersensitivity reactions including insect bites reaction and urticaria as well as adverse cutaneous drug eruption. Among the infections, viral infections were diagnosed in 1134 patients (45.95%) and bacterial infections in 990 patients (40.11%) accounting for majority of infections. Fungal infections were present in only 344 patients (13.94%). Scabies was the most common infestation which was noted in 579 (7.5%) patients. Both infections (2.03 : 1) and scabies (1.75 : 1) were more in males compared to the females.

Table 1 summarizes all the diseases among males and females in the one year period.

According to the age group distribution, age group of 1–5 years recorded almost half of the total consultations with 3770 (49.07%) patients, whereas neonates group had the least number of only 58 (0.75%) patients (Table 2).

Table 2 describes in detail the various diseases according to the categories of diagnosis. Among others, majority of patients 137 (43.2%) had nonspecific papular eruptions, 66 (20.82%) had localized pruritus, 18 (5.675) had generalized pruritus, and erythematous rash was present in 11 (3.4%) patients, where an exact diagnosis could not be reached.

Insect bite reaction was diagnosed in 847 patients (males = 520, females = 327). Similarly, urticarial eruption was noted in a total of 606 (7.89%) patients with 375 males and 231 females. Adverse cutaneous drug eruption was present in 57 patients (0.074%) but the severe forms of drug eruption such as Steven-Johnsons syndrome (SJS) were present only in 2 cases (0.02%).

Scabies was present in 580 (7.53%) of the patients and more common in males (*M* = 369, *F* = 210). Miliaria, a common disorder of sweat glands, was present in 493 patients (6.41%) of all cases with 296 males and 197 females. It was found to be more common in infants age group as compared to others. Similarly, acne vulgaris, a common problem in adolescents, was present in only 11 female patients (0.01%). Congential nevus was present in 78 (97.5%) patients whereas acquired nevus was diagnosed in only 2 (2.5%) patients.

Eczematous condition was the most common diagnosis in all age groups, except in the neonatal period where miliaria was the most common.

**Figure 1:**
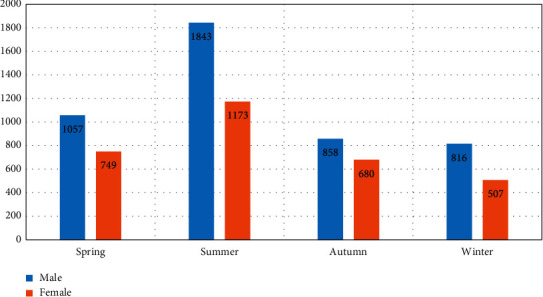
Bar diagram showing the seasonal variation of skin diseases among different sex.

The highest number of patients was noted during summer months (*n* = 3016) (39.26%) with a total of 1843 (23.99%) males and 1173 (15.27%) females. The lowest number of patients was recorded during the winter months with a total of 1323 patients (17.22%), consisting of 816 (10.62%) males and 507 (6.59%) females. The distribution of patients over the four different seasons is shown in Figure 1.

**Figure 2:**
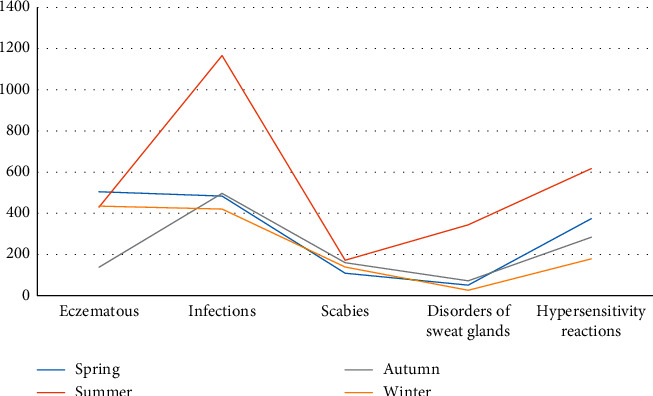
Distribution of common skin diseases according to the seasons.

Eczema and viral infection were the most common causes throughout the year. The distribution of the common diseases over the seasonal variation is shown in Figure 2.

**Figure 3:**
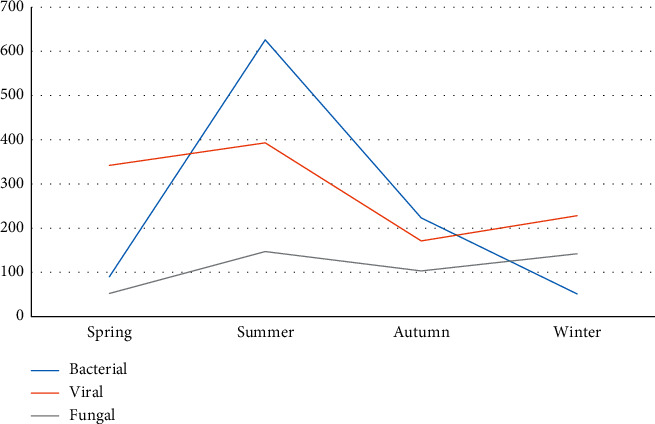
Seasonal variation of different skin infections in the pediatric patients.

Similarly, bacterial infections were more common during the summer months as compared to other seasons (Figure 3). However, skin diseases (both infectious and noninfectious) are significantly more common during warm periods (summer and spring) months as compared to cold (autumn and winter) months.

Genodermatoses, nutritional dermatoses, and genetic blistering disease were the least common conditions diagnosed in the pediatric OPD.

## 4. Discussion

There is a general lack in available data regarding the pediatric dermatoses in Nepal with only a handful of published literature dedicated to pediatric dermatoses. This study was conducted in Nepal's only tertiary care central level pediatric referral hospital located in Kathmandu. We looked into the prevalence of pediatric dermatoses as well as the pattern of skin diseases according to the different pediatric age groups and the variation according to the seasons.

This study had the largest number of data as compared to the previous studies on pediatric dermatoses from the country.

There was a significant variation of dermatosis according to the different age groups. However, the most common dermatosis in our study was infectious dermatoses followed by eczema. Other studies from Nepal have shown that infections and eczema were also the two most common conditions [3, 4, 8, 9]. Similar results were also noted from several studies from India [2, 10–12]. In this study, we found higher prevalence of pediatric skin diseases among males as compared to females (1.47 : 1) which was consistent with other studies conducted in Nepal and abroad [3, 4, 10, 12, 13]. But, a more common female preponderance was noted in a study from Turkey by Tamer et al. [14]. This significant difference might be due to increased outdoor activities of male children as compared to female children.

The age group of 1–5 years had almost half (49.7%) of the total patients (*n* = 3770). This result was consistent with other studies conducted inside as well as outside Nepal [3, 4, 10].

Among the infections, viral infection was the most common followed by bacterial and fungal infections which was similar to the study conducted in Turkey [15]. However, few other studies had revealed bacterial infections to be more common in contrast to ours [4, 11, 16]. Similarly, Poudyal et al. [3] and Kirprono et al. [17] found dermatophytosis as the most common dermatosis. On the other hand, a study from Turkey had lower prevalence of infections but a higher prevalence of acne [14].

Pattern of pediatric dermatoses varied according to the season. In our study, bacterial infection and miliarial eruption were more common during the summer months which was similar to studies conducted by Banerjee et al. [16] and Shrestha et al. [4]. Spring and autumn showed a high prevalence of eczema which was the commonest dermatoses. Certain studies found a significant rise in scabies during winter season [3, 10], but our study failed to recognize such association but rather scabies was more common during summer months. Similarly, fungal infections were common even during the winter months. The hot and humid condition during summer season might be the reason for the increase in bacterial infections and miliaria.

The overall prevalence of scabies (7.53%) in our study was slightly greater than that of Poudyal et al. (4.4%) [3], Shrestha et al. (5.03%) [4], Reddy et al. (3.6%) [12], Patel et al. (5.32%) [13], Afsar (0.4%) [18], Tamer et al. [14], and Sacchidanand et al. (6.97%) [10], but lower than that of Banerjee et al. [16] in India and Shrestha and Mikrani [9] in Nepal. Studies from Turkey had a very low incidence of scabies as compared to the subcontinent [14, 18]. The lack of proper sanitation and poor hygiene might be the reason for the increased number of scabies.

There were only a couple of cases of Steven-Johnson syndrome (0.02%) recorded in our study. The possibility that the sick patients are taken care of in the emergency room rather than in out-patient department resulted in such findings.

## 5. Conclusion

Overall, the two most common pediatric dermatoses include infections and eczema. The dermatoses varied according to the different age groups and different seasons. This study showcased the burden of pediatric dermatoses in the country. The large disease burden shown in this study should persuade policy makers in improving the dermatological services offered in such institutes which are scarce currently.

Furthermore, starting residency rotations and recognizing the subspecialty are necessary in the country to excel in this field of pediatric dermatology.

## Figures and Tables

**Table 1:** Distribution of diseases according to different categories in a one year period.

Disease categories	Males	Females	Total
Infections	1466 (19.08%)	1002 (13.04%)	**2468 (32.12%)**
Eczematous disorders	1006 (13.09%)	705 (9.18%)	1711 (22.27%)
Hypersensitivity disorders	935 (12.17%)	575 (7.48%)	1510 (19.65%)
Infestations	370 (4.82%)	218 (2.84%)	588 (7.65%)
Disorders of sweat glands	296 (3.85%)	197 (2.56%)	493 (6.42%)
Others, uncategorized	164 (2.13%)	153 (1.99%)	317 (4.13%)
Pigmentary disorders	51 (0.66%)	48 (0.62%)	99 (1.29%)
Blood and vascular disorders	41 (0.53%)	48 (0.62%)	89 (1.16%)
Xerosis	62 (0.81%)	27 (0.35%)	89 (1.16%)
Papulosquamous disorder	56 (0.73%)	31 (0.40%)	87 (1.13%)
Nevi	37 (0.48%)	43 (0.56%)	80 (1.04%)
Disorders of hair	24 (0.31%)	20 (0.26%)	44 (0.57%)
Disorders of keratinizations	22 (0.29%)	15 (0.20%)	37 (0.48%)
Disorders of sebaceous glands	13 (0.17%)	11 (0.14%)	24 (0.31%)
Disorders of mucous membrane	12 (0.16%)	5 (0.07%)	17 (0.22%)
Disorders of nail	6 (0.08%)	3 (0.04%)	9 (0.12%)
Blistering diseases (inherited)	5 (0.07%)	1 (0.01%)	6 (0.08%)
Genodermatoses	3 (0.04%)	3 (0.04%)	6 (0.08%)
Nutritional dermatoses	4 (0.05%)	0 (0.00%)	4 (0.05%)
Total	4574 (59.53%)	3109 (40.47%)	7683 (100%)

**Table 2:** Patients' distribution according to sex and various age groups.

Age group	Male (%)	Female (%)	Total (%)
1–28 days	39 (0.51)	19 (0.25)	58 (0.75)
28 days–1 year	1159 (15.09)	752 (9.79)	1911 (24.87)
2–5 years	2306 (30.01)	1464 (19.06)	**3770 (49.07)**
6–11 years	878 (11.43)	741 (9.64)	1619 (21.07)
11–14 years	192 (2.50)	133 (1.73)	325 (4.23)
Total	4574 (59.53)	3109(40.47)	7683 (100)

## Data Availability

Data are available from the corresponding author upon request.
